# Gene and transcript expression patterns, coupled with isoform switching and long non-coding RNA dynamics in adipose tissue, underlie the longevity of Ames dwarf mice

**DOI:** 10.1007/s11357-024-01383-x

**Published:** 2024-10-15

**Authors:** Sebastian Cano-Besquet, Maiyon Park, Nadia Berkley, Michelle Wong, Sarah Ashiqueali, Sarah Noureddine, Adam Gesing, Augusto Schneider, Jeffrey Mason, Michal M. Masternak, Joseph M. Dhahbi

**Affiliations:** 1 Department of Medical Education, School of Medicine, California University of Science & Medicine, Colton, CA USA; 2 University of Florida, Gainesville, FL 32611 USA; 3 College of Medicine, Burnett School of Biomedical Sciences, University of Central Florida, Orlando, FL USA; 4 Department of Endocrinology of Ageing, Medical University of Lodz, Lodz, Poland; 5 Faculdade de Nutrição, Universidade Federal de Pelotas, Pelotas, Brazil; 6 College of Veterinary Medicine, Department of Veterinary Clinical and Life Sciences, Center for Integrated BioSystems, Utah State University, Logan, UT USA; 7 Department of Head and Neck Surgery, Poznan University of Medical Sciences, Poznan, Poland

**Keywords:** Ames Dwarf mice, Longevity, Adipose, Transcriptome, Long non-coding RNAs, Isoform switching

## Abstract

Our study investigates gene expression in adipose tissue of Ames dwarf (df/df) mice, whose deficiency in growth hormone is linked to health and extended lifespan. Recognizing adipose tissue influence on metabolism, aging, and related diseases, we aim to understand its contribution to the health and longevity of df/df mice. We have identified gene and transcript expression patterns associated with critical biological functions, including metabolism, stress response, and resistance to cancer. Intriguingly, we identified genes that, despite maintaining unchanged expression levels, switch between different isoforms, impacting essential cellular functions such as tumor suppression, oncogenic activity, ATP transport, and lipid biosynthesis and storage. The isoform switching is associated with changes in protein domains, retention of introns, initiation of nonsense-mediated decay, and emergence of intrinsically disordered regions. Moreover, we detected various alternative splicing events that may drive these structural alterations. We also found changes in the expression of long non-coding RNAs (lncRNAs) that may be involved in the aging process and disease resistance by regulating crucial genes in survival and metabolism. Through weighted gene co-expression network analysis, we have linked four lncRNAs with 29 genes, which contribute to protein complexes such as the Mili-Tdrd1-Tdrd12 complex. Beyond safeguarding DNA integrity, this complex also has a wider impact on gene regulation, chromatin structure, and metabolic control. Our detailed investigation provides insight into the molecular foundations of the remarkable health and longevity of df/df mice, emphasizing the significance of adipose tissue in aging and identifying new avenues for health-promoting therapeutic strategies.

**Supplementary Information:**

The online version contains supplementary material available at 10.1007/s11357-024-01383-x.

## Introduction

Ames dwarf mice (df/df) exhibit extended lifespans and resistance to age-related diseases due to growth hormone (GH) deficiency and reduced insulin/IGF-1 signaling [1–6]. These unique traits establish these mice as an invaluable model organism for aging and longevity research. Their longevity and healthspan are attributed to altered gene expression across various tissues, reflecting the impact of suppressed GH and IGF-I signaling and changes in metabolic processes, including amino acid metabolism, the TCA cycle, mitochondrial function, and lipid metabolism [7–11]. Additionally, df/df mice demonstrate reduced apoptosis, enhanced cellular longevity [8], delayed ovarian aging, and decreased expression of inflammation genes [12, 13]. Furthermore, miRNA and small non-coding RNA profiles support a youthful ovarian state, contributing to their longevity and cancer resistance [12–14]. Thus, the df/df mutation triggers a range of changes in gene expression, which seem to play a crucial role in extending lifespan and slowing down the aging process in these mice [9–11].

Adipose tissue is recognized as a crucial metabolic regulator relevant to the pathology of age-related diseases [3, 4, 15, 16]. In df/df mice, reduced fat mass is linked to slowed aging and longer lifespans, highlighting the role of body composition in aging [17–19]. Particularly, brown adipose tissue’s influence on insulin sensitivity, energy metabolism, and thermogenesis is essential for the longevity observed in these mice [20, 21]. Our recent study indicates a rise in fibroadipogenic precursors and stem cells in df/df visceral fat, with high miR-449a levels—a molecule typically reduced with age—contributing to lowered inflammation and decelerated cellular aging [22]. Moreover, miR-449a’s suppression of senescence markers emphasizes the adipose tissue’s significance in modulating cellular aging and extending lifespan [23, 24].

Recognizing adipose tissue’s significant influence on aging, metabolism, and lifespan, as well as the insights gene expression profiling provides, our study aimed to elucidate these complex processes through a comprehensive transcriptomic analysis of df/df mouse adipose tissue. We used RNA sequencing paired with bioinformatics tools to identify specific gene expression changes. Our findings include a range of differentially expressed transcripts and genes, which encompass both protein coding RNAs and lncRNAs. Through functional enrichment analyses, we explored their biological significance. We also examined the regulatory roles of lncRNAs in relation to protein-coding genes using weighted gene co-expression network analysis (WGCNA). Additionally, isoform switching analysis revealed new aspects of gene expression regulation, which may offer insights into their contributions to longevity and healthspan. This comprehensive view of transcriptomic alterations could inform the development of targeted therapies for aging-related conditions.

## Materials and methods

### Library construction for RNA sequencing and subsequent bioinformatics analysis

Heterozygous females with a normal phenotype (N/df) were crossed with homozygous Ames dwarf males (df/df) to produce offspring that were either heterozygous (N/df) or homozygous dwarf (df/df). The heterozygous (N/df) offspring carry one normal *Prop1* allele and one mutant *df* allele but are phenotypically normal and exhibit gene expression profiles similar to homozygous wild-type (N/N) mice. Therefore, they serve as appropriate littermate controls for the dwarf (df/df) mice in our study. All mice were maintained under controlled temperature and light conditions and were provided nutritionally balanced diet (Rodent Laboratory Chow 5001) ad libitum. For RNA sequencing, male offspring aged 8 to 12 months were divided into control (N/df, *n* = 4) and dwarf (df/df, *n* = 5) groups [22]. Following an overnight fast, the mice were anesthetized with 2.5% isoflurane and euthanized for visceral fat tissue collection. The harvested tissues were immediately snap-frozen on dry ice and stored at − 80 °C. RNA was extracted from 30 mg visceral fat tissue using RNeasy Mini Kit (QIAGEN, Hilden, Germany), quantified, and utilized to prepare sequencing libraries with the Illumina NEBNext Ultra Directional RNA Library Prep Kit. Sequencing was carried out on an Illumina HiSeq 2000 system at the University of California Riverside Genomics Core, generating 100-base pair paired-end reads.

Sequencing data quality was first evaluated with FASTQC, followed by adaptor sequence removal using Trimmomatic [25]. Reads were then aligned to the mouse reference genome (GRCm39, GENCODE M27) with HISAT2 (v2.2.1) [26], and StringTie [27] was used for transcript assembly and expression quantification, marking novel transcripts with an “MSTRG” prefix. This resulted in gene and transcript count matrices for differential expression analysis.

Differential expression of mRNA and lncRNA was analyzed with DESeq2 (v1.40.2) [28], focusing on significant isoform expression differences. Criteria for differential expression included |log2 fold change|> 0.58 and adjusted *p*-value < 0.05.

### Isoform switching analysis

In our study, we used IsoformSwitchAnalyzeR [29] to analyze isoform switching in the adipose tissue of df/df mice as compared to the control group (N/df). The analysis began by inputting the isoform expression data, acquired from StringTie, into IsoformSwitchAnalyzeR, which was further supplemented with exon annotations for each isoform. The primary metric was the isoform fraction (IF)—a measure of the proportion of a gene’s total expression that is accounted for by each individual isoform. The difference in IF between the df/df group and the control group (N/df) was computed as dIF = IF_df/df – IF_N/df. An isoform was identified as being significantly switched-up in df/df if dIF > 0.10 and FDR < 0.05. Conversely, an isoform was deemed significantly switched down in df/df if dIF <  − 0.10 and FDR < 0.05.

Further analysis of switched isoforms focused on their potential impact on gene function, considering intron retention (IR), alterations in amino acid sequences of open reading frames (ORFs), susceptibility to nonsense mediated decay (NMD), and changes in protein domain structures [30]. Detailed analysis of significant isoform switches utilized various bioinformatics tools: IUPred2A [31] for assessing intrinsically disordered regions, SignalP [32] for signal peptide prediction, CPC2 [33] for coding potential evaluation, and Pfam [34] for analyzing protein domains. Additionally, the study investigated alternative splicing events influencing isoform switching, revealing potential modifications in gene function through changes in transcription start and termination sites, as well as exon junctions.

### Weighted gene co-expression network analysis (WGCNA) of protein-coding mRNAs and long non-coding RNAs (lncRNAs)

WGCNA was used to identify gene clusters, or modules, that demonstrate similar expression patterns by establishing a weighted gene expression network [35]. The strength of this network’s internal connections, or adjacencies, were assessed in conjunction with their association to particular traits via correlation analysis of gene expression profiles. Our study used differentially expressed protein-coding mRNAs and lncRNAs to construct a weighted correlation network. Prior to the WGCNA, we normalized the gene count matrix provided by StringTie and applied variance stabilization using the vst() function from the DESeq2 package. The choice of an optimal power parameter, which is essential for precise module identification, was determined by the pickSoftThreshold() function in WGCNA. This function graphs the scale-free fit index (signed *R*^2^) against a range of power values, thereby ensuring the selection of a threshold that enables a scale-free topology, indicative of biological networks. In our analysis, a soft-thresholding power (*β*) of 16 was selected. This power produced a network characterized by a signed *R*^2^ value above 0.80, under the default parameter settings.

After creating a gene co-expression network and using hierarchical clustering, a dendrogram sorted genes into color-coded modules, with uncorrelated genes falling into a “Gray” module. We focused on module-trait associations by correlating the eigengenes of each module with the df/df trait. This approach aimed to identify modules significantly associated with the df/df phenotype. Gene significance (GS) was determined by the absolute value of the correlation between gene expression and the trait, signifying the gene’s pertinence to the trait. Conversely, module membership (MM) was quantified by the correlation of a gene’s expression with its respective module eigengene, reflecting its importance within the module. By analyzing modules that exhibited strong correlations with the df/df trait and identifying genes within those modules with significant GS and MM values, we could detect key genes and pathways potentially impacting the df/df phenotype.

### Functional enrichment analysis

Functional enrichment analysis utilized gprofiler2 to assess gene functions and pathways [36], examining overrepresentation in various databases through a hypergeometric test. With “domain_scope” set to “annotated,” analysis prioritized genes with known annotations, sorted by fold change significance. Adjustments for false discovery rates were made to validate the results, with significant enrichment outcomes (FDR < 0.05) visualized using ggplot2 in R. The functional enrichment analysis included differentially expressed genes, targets of lncRNAs, and mRNAs co-expressed with lncRNAs, with mRNA-lncRNA interactions predicted using RNAInter [37].

## Results

### Multi-level RNA-Seq analysis elucidates gene expression dynamics in adipose tissue of df/df mice

**Fig. 1:**
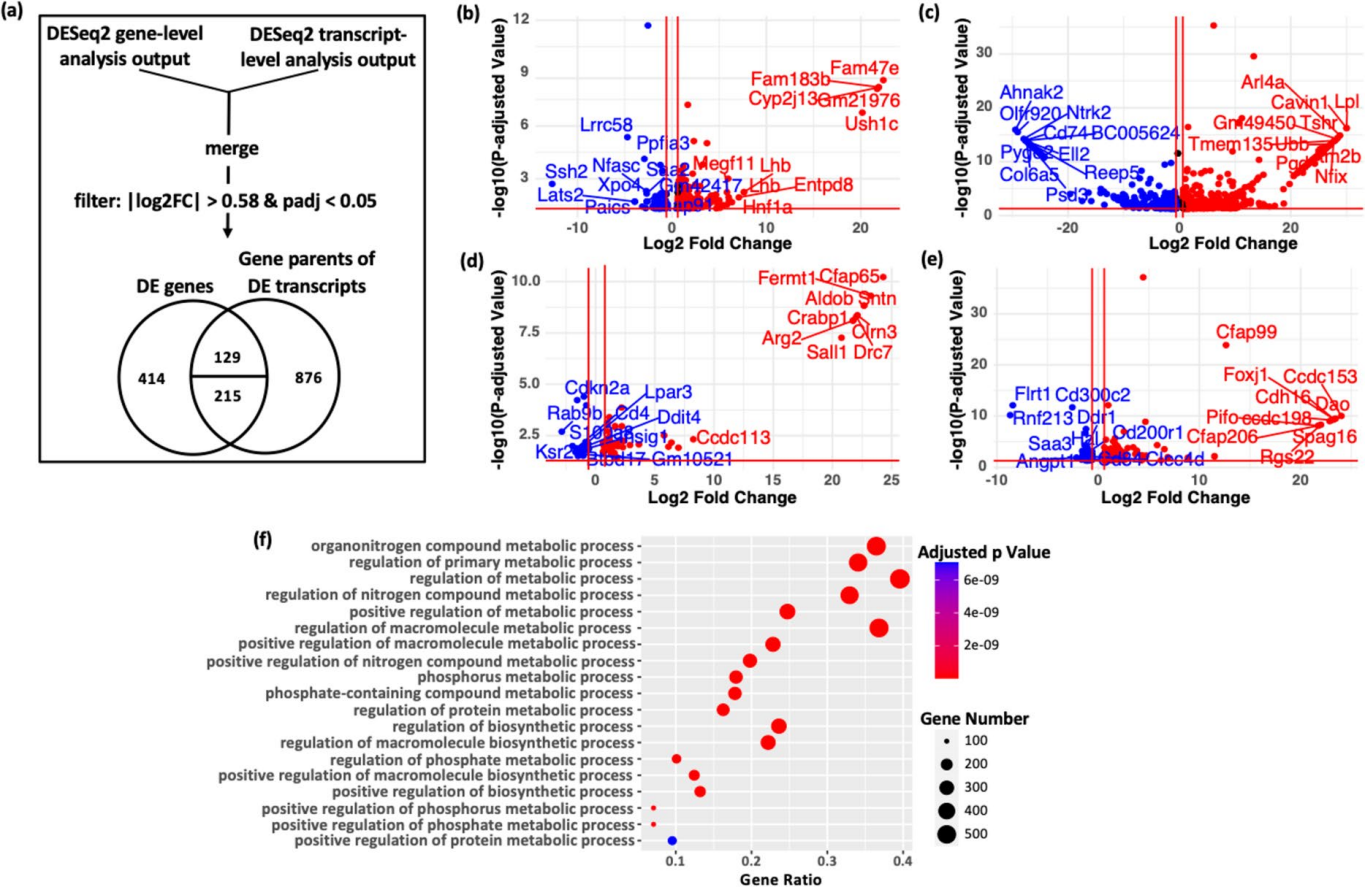
Differential gene and transcript expression in df/df mouse adipose tissue. **a** RNA-Seq workflow overview. A schematic representation of the RNA sequencing analysis pipeline applied to adipose tissue from df/df mice. The analysis initiates with the assessment of differential expression at both gene and transcript levels. Identified differentially expressed (DE) entities are further processed to distinguish those altered exclusively at the gene or transcript level, as well as those impacted at both levels. A Venn diagram illustrates the distribution of differential expression: 414 genes at the gene level only, 876 at the transcript level only, 129 at both levels, and 215 genes exhibiting gene-level DE with a subset of their transcripts also differentially expressed. **b–e** Volcano plots of differential expression. Graphical representations of gene expression changes across different categorizations. **b** Four hundred fourteen genes with exclusive gene-level changes, **c** 876 genes with transcript-level changes only, **d** 129 genes with concurrent gene and transcript-level changes, and **e** 215 genes with gene-level changes with a subset of their transcripts also changed. Upregulated genes are marked with red dots, while blue dots denote downregulated genes. **f** Gene Ontology biological processes enrichment. Visualization of shared enriched biological processes for the gene expression categories described in (**a**). Enrichment analysis conducted using gprofiler2, employing the false discovery rate (“fdr”) correction with a significance cutoff of *p* < 0.05. The graph illustrates non-redundant, enriched biological terms, ordered by significance (adjusted *p*-value) on the *y*-axis. The *x*-axis shows the enrichment ratio, indicating the observed over expected gene frequency within a pathway. Dot sizes represent the count of implicated genes (intersection size), and the color intensity indicates the adjusted *p*-value’s significance

The study began by evaluating the impact of the df/df genotype on gene expression within adipose tissue. Adipose tissue samples from both df/df mice and their normal littermates were analyzed using RNA sequencing. DESeq2 was employed to quantify gene and transcript expression levels. Selection criteria for further analysis included a “protein_coding” biotype, a |log₂ fold change (FC)|> 0.58, and an adjusted *p*-value < 0.05. Where differential expression was noted only at the transcript level, the associated parent genes were also considered (Fig. 1a).

To elucidate the transcriptional dynamics associated with the df/df genotype, the genes were classified into four categories based on their patterns of expression changes:

- 414 genes with changes at the gene level only (Fig. 1a and Table S1).
- 876 genes with changes at the transcript level only (Fig. 1a and Table S2).
- 129 genes with changes at both gene and transcript levels (Fig. 1a and Table S3).
- 215 genes with changes at the gene level and a subset of their transcripts (Fig. 1a and Table S4).

Volcano plots (Fig. 1b–e) were used to visually represent the gene expression differences within these categories. This layered analysis of RNA-Seq data enables the identification of subtle changes in gene expression, thereby enhancing the understanding of how the df/df genotype influences adipose tissue.

### Assessing the biological relevance of multi-level RNA-Seq analysis

Functional enrichment analysis [36] of differentially expressed genes in Fig. 1a and Tables S1**–**S4 showed unique associations with biological processes. Genes altered at the gene level were linked to cell communication, motility, migration, and intercellular signaling pathways (Figure S1a), while changes at the transcript level corresponded to cell cycle, apoptosis, MAPK cascade, and stress response pathways (Figure S1b). Genes with both gene and transcript level changes were associated with immune and inflammatory responses (Figure S1c). Alterations at the gene level and in some transcripts were associated with cell adhesion, various signaling pathways, and fat cell differentiation (Figure S1d). Finally, common genes across all groups impacted metabolic processes including metabolism of proteins, nitrogen, and organonitrogen compounds (Fig. 1f). The observed differences in expression at the gene or transcript level, or both, appear to have a significant impact on specific cellular functions. Further research is necessary to understand the link between these gene expression patterns and their possible effects on the health and longevity of df/df mice.

### Analysis of isoform switching in genes with unchanged overall expression reveals further complexity in the transcriptome of df/df mice, suggesting additional regulatory layers

**Fig. 2:**
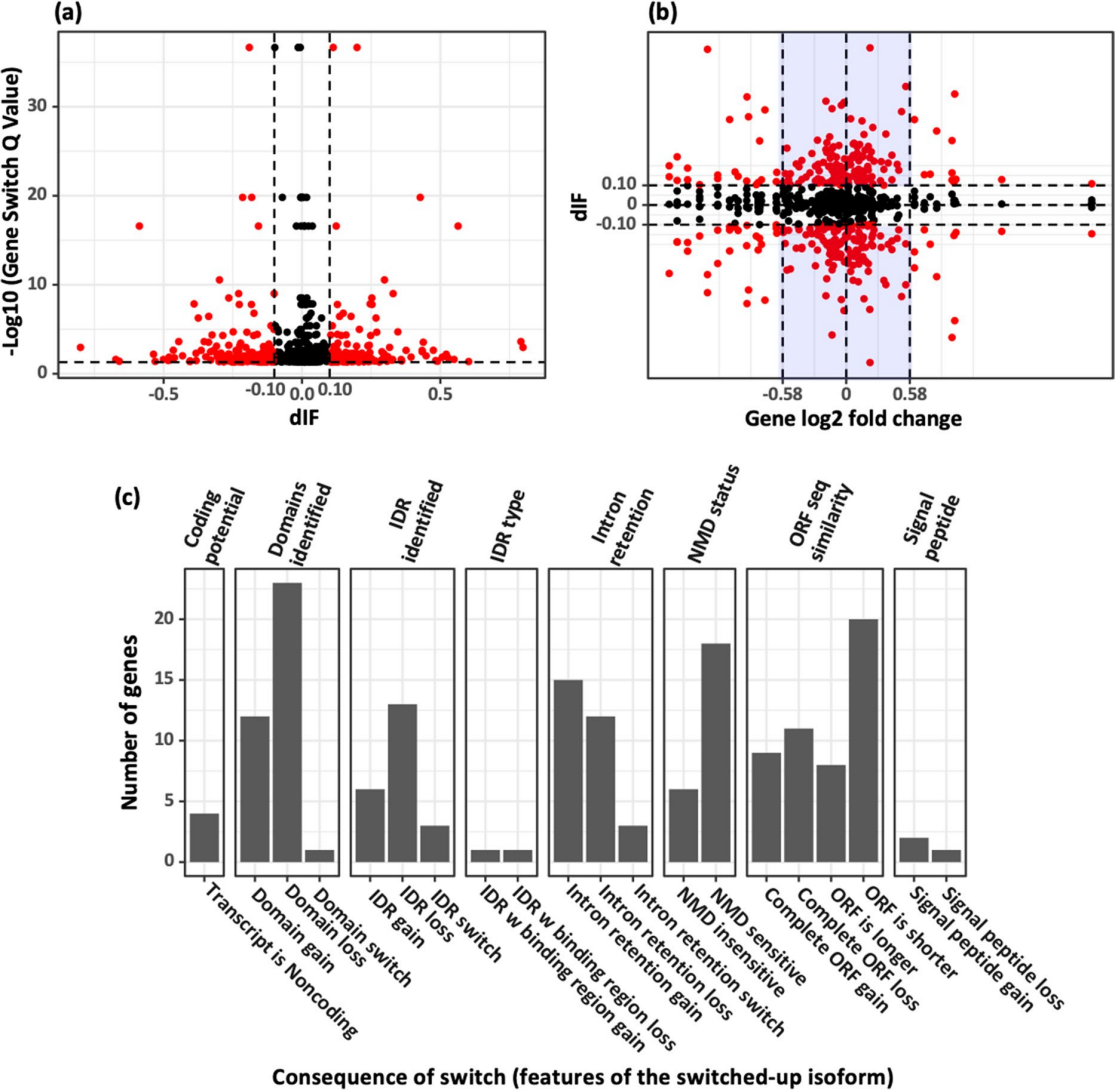
Isoform switching in gene expression. **a** Isoform switching volcano plot. Analysis of isoform usage changes in df/df mice. The graph plots isoform fraction difference (dIF) against statistical significance (“gene switch *Q* value”). Significant isoform switches are defined by |dIF|> 0.1 and a gene switch *Q* value < 0.05 and are indicated in red. The vertical dashed lines represent the |dIF| threshold of 0.1, and the horizontal dashed line marks the gene switch *Q* value threshold of 0.05. **b** Correlation of isoform switching with gene expression changes. The volcano plot presents the relationship between gene expression change (“Gene log2 fold change”) on the *x*-axis and dIF on the *y*-axis. Significant isoform changes (red dots) meet the criteria of |dIF|> 0.1 and gene switch *Q* value < 0.05. The light blue shaded area highlights genes without gene-level expression changes (|Gene log2 fold change|< 0.58 and “gene_q_value” > 0.05) yet significant in isoform switching. Dashed lines indicate dIF thresholds at ± 0.1 and Gene log2 fold change thresholds at ± 0.58. **c** Functional consequences of isoform switching in genes with unchanged expression levels. The bar graph categorizes the various functional consequences resulting from isoform switching in genes whose overall expression remains unchanged. On the *x*-axis, the categories of functional consequences are listed, with the corresponding functional attributes detailed above each bar. These categories distinguish between the effects of isoforms that are switched-up versus those that are switched-down. The *y*-axis shows the count of genes manifesting each type of functional consequence related to isoform switching indicated on the *x*-axis

We demonstrated that a comprehensive analysis combining gene and transcript levels yields a fuller understanding of differential expression. By further incorporating isoform switch analysis [29], which detects changes in isoform usage even when overall gene expression is unchanged, we enhanced our evaluation of gene expression in df/df mouse adipose tissue. We found that specific isoforms are switched-up or switched-down independent of the gene’s total expression. This is indicated by an isoform fraction difference |dIF|> 0.1, and a gene switch Q value < 0.05 (Fig. 2a). Despite no overall change in gene expression, 100 genes showed significant isoform switching, affecting 124 isoforms (Fig. 2b and Table S5). This indicates an additional dimension of gene expression regulation that could influence df/df mice health and longevity.

### Isoform switching in genes with unchanged overall expression levels is associated with diverse functional consequences

To assess the potential functional consequences of isoform switching in genes with unchanged overall expression, we examined features including protein domains, intron retention (IR), intrinsically disordered regions (IDRs), coding potential, open reading frame (ORF) lengths, and susceptibility to nonsense-mediated decay (NMD) by comparing isoforms that switched up with those that switched down. Our study of 100 genes uncovered that 65 exhibited significant isoform switches with distinct functional features, as detailed in Table S6. We noted prevalent isoform switches affecting protein domains, IDRs, IR, NMD sensitivity, and ORF lengths (Fig. 2c). Genes with switched-up isoforms frequently presented with loss of domains and IDRs, increased IR, and shortened ORFs. While these alterations did not affect the overall gene expression levels, they could have substantial impacts on the proteins’ functions. This insight enhances our understanding of the molecular factors that may contribute to the lifespan of df/df mice and guides future research directions.

### Alternative splicing is associated with isoform switching in genes with unchanged overall expression

**Fig. 3:**
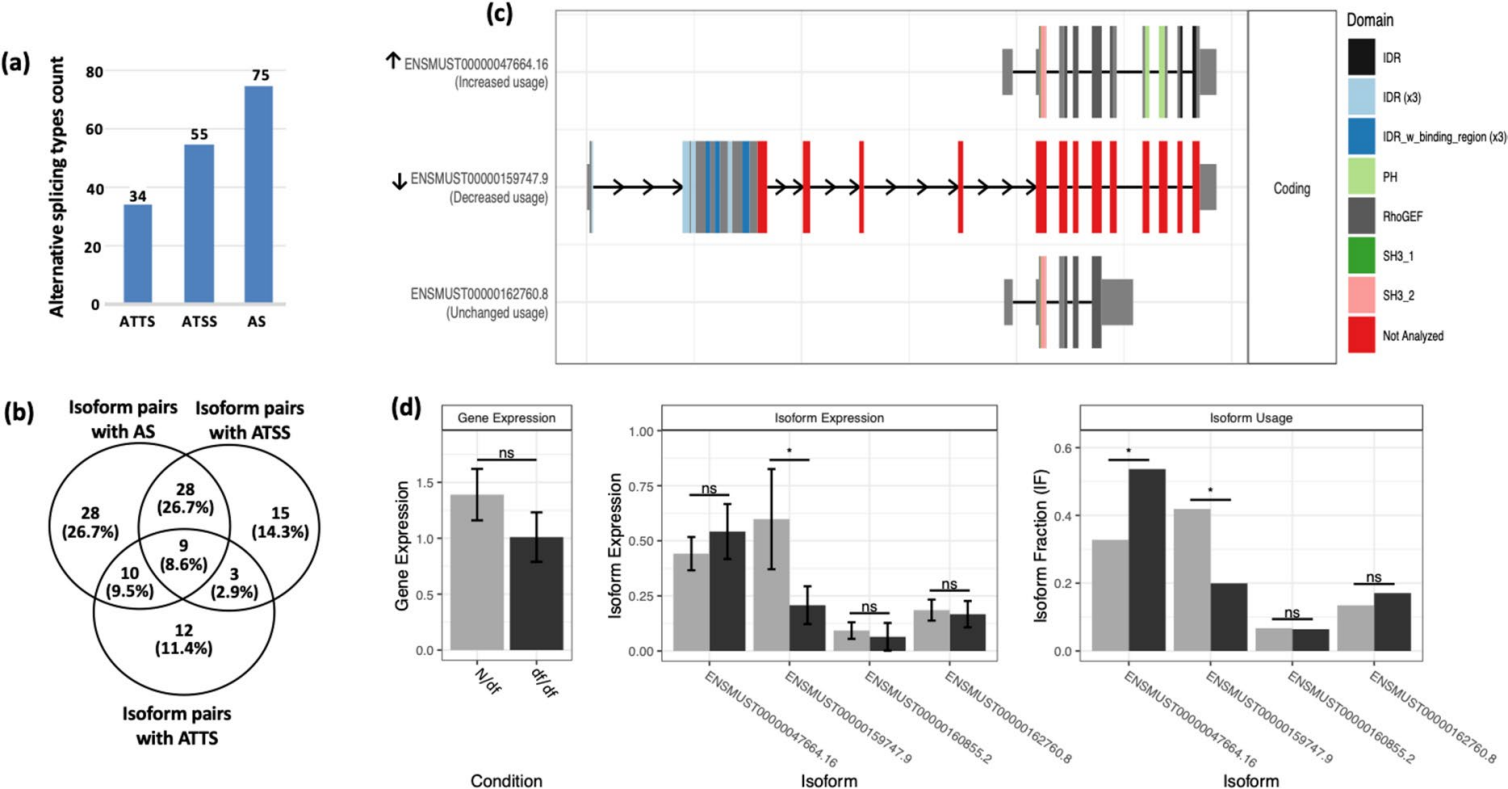
Splicing mechanisms in genes maintain unchanged expression but undergo isoform switching. **a** Distribution of alternative splicing mechanisms. A bar chart showing the frequency of different splicing events: alternative transcription start sites (ATSS), alternative transcription termination sites (ATTS), and alternative splicing (AS), the latter relating to variations in exon-exon junctions and intron retention. The frequency of these events is measured, suggesting their potential role in isoform switching that leads to functional diversity without altering overall gene expression levels. **b** Comparative analysis of splicing events. The Venn diagram illustrates the distribution of 105 isoform pairs, indicating the presence of ATSS, ATTS, or AS events in isolation or combination. This demonstrates the complexity of splicing mechanisms contributing to the functional outcomes of isoform switching in the backdrop of unchanged gene expression. **c–d** Isoform switching in the Arhgef4 gene. **c** Arhgef4 isoform structures. The graph shows the structural differences between Arhgef4 gene isoforms. Exons are depicted as solid blocks, introns as connecting lines, and 5′ and 3′ UTRs as narrower blocks at the transcript’s ends. Distinctive protein domains are highlighted in color. Arrows indicate the two contrasted isoforms identified by Ensembl transcript IDs, with the upward arrow for the “switched-up” isoform (ENSMUST00000047664.16) featuring a shorter open reading frame (ORF) with the absence of intrinsically disordered regions (IDRs), and the downward arrow for the “switched-down” isoform (ENSMUST00000159747.9) preserving a longer ORF with IDRs. **d** Expression analysis of Arhgef4. The graph contrasts unchanged overall gene expression (left panel) with isoform-specific expression changes (middle panel) and differential usage of “switched-up” versus “switched-down” isoform pairs (right panel). “ns” denotes non-significant differences; asterisks indicate an adjusted *p*-value < 0.05. N/df refers to mice that are heterozygous for the df allele, exhibit a normal phenotype, and are used as littermate controls for the dwarf mice (df/df)

To investigate a potential association between alternative splicing and isoform switching in genes that maintain unchanged overall expression levels, we analyzed their splicing patterns. This analysis specifically addressed alternative transcription start sites (ATSS), alternative transcription termination sites (ATTS), and general alternative splicing (AS) events. The latter mainly involves changes in intron structure, such as differences in exon-exon junctions. Our analysis of 100 genes listed in Table S5 identified 94 genes that showed splicing differences across 105 isoform pairs (Table S7). Among these isoform pairs, there were 34 involving ATTS, 55 with ATSS, and 75 with AS changes (Fig. 3a). A Venn diagram (Fig. 3b) categorizes these 105 isoform pairs by their differential splicing events: 28 pairs exclusively had AS changes, 15 had only ATSS changes, and 12 had only ATTS changes. Nine pairs underwent all three types of splicing events simultaneously. The rest had a combination of two out of the three splicing event types: ATSS and AS, ATSS and ATTS, or ATTS and AS.

Lastly, we matched the functional consequences of isoform switching (outlined in Table S6) with the types of splicing events (listed in Table S7). We found that 60 genes across 64 isoform pairs exhibited functional consequences that are associated with AS, ATSS, ATTS, or their combinations (Table S8). These findings suggest that genes are capable of alternative splicing, potentially leading to isoform switching and subsequent alterations in function, without requiring changes in the overall expression levels of the genes.

### Analyzing isoform switching unveils links to decreased cancer susceptibility and improved metabolism, aligning with the enhanced health and increased longevity in df/df mice

Isoform switching analysis operates on the premise that an increase in one isoform’s usage within a gene is counterbalanced by a decrease in another isoform, resulting in unchanging overall gene expression. Guided by this principle, we established rigorous criteria to identify functionally significant isoform switches in genes where overall expression does not change: (a) finding at least two isoforms per gene with significant switching, (b) the presence of opposite patterns of usage between the isoforms—one showing switched-up and the other switched-down usage, (c) identifying a significant functional difference between isoform pairs, and (d) ensuring at least one isoform’s expression remains unchanged at the transcript level, adding significance to differential isoform usage against a backdrop of unchanged overall gene expression. Application of these criteria revealed seven genes with unchanged overall expression but significant isoform usage changes. Notably, these genes were not previously associated with df/df mouse models, thus demonstrating the advanced detection capability of isoform switching analysis.

In a function analysis of the seven genes, three (Arhgef4, PHF3, and Zfp607a) were found to play a role in cancer. Arhgef4’s isoform switch, causing loss of an IDR and a shorter ORF (Fig. 3c, d), may affect cancer progression by altering cell migration and the APC tumor suppressor’s function, potentially impacting cancer, and lifespan in df/df mice. For PHF3, one isoform lacks the ORF (Figure S2), and the less used isoform with IDRs and domains important for transcription and mRNA stability could be linked to glioblastoma survival rates; its decreased usage might be associated with lower df/df mice cancer rates. In Zfp607a, the switch to an NMD-sensitive isoform lowers functional Zfp607a levels (Figure S3), which might correlate with reduced tumor incidence in df/df mice due to its ties to hepatocellular carcinoma prognosis.

Isoform switches have been noted in three metabolism-related genes: Slc25a25, Dot1l, and Snap47. The Slc25a25 isoform switch involves the loss of an ORF and key domains (Figure S4). The less-used isoform retains domains (EF-hand and Mito_car) critical for mitochondrial ATP transport and obesity resistance, potentially affecting the metabolic efficiency in df/df mice. For Dot1l, the isoform switch alters the TSS, leading to decreased intron retention (Figure S5), indicating a shift to a functional state. Dot1l plays a critical role in regulating lipid biosynthesis within macrophages; therefore, this isoform change may influence both lipid biosynthesis and inflammation, potentially conferring protection against metabolic diseases in df/df mice. Snap47’s switched-up isoform has an intact ORF and upstream TTS (Figure S6), playing a role in lipid droplet fusion and transport; variations here could impact transcript stability and abundance which might influence lipid metabolism and storage in df/df mice.

Finally, the gene Mid1-ps1 has a switched-up isoform with a longer ORF and new domains, unlike its switched-down counterpart which is prone to NMD due to a premature termination codon (Figure S7). Contrary to its classification as an unprocessed pseudogene, Mid1-ps1 may encode a functional protein that is significant for cellular functions in df/df mice, based on IsoformSwitchAnalyzeR analysis.

### Differentially expressed lncRNAs in df/df mice regulate key pathways impacting metabolism, cell signaling, and stress response

**Fig. 4:**
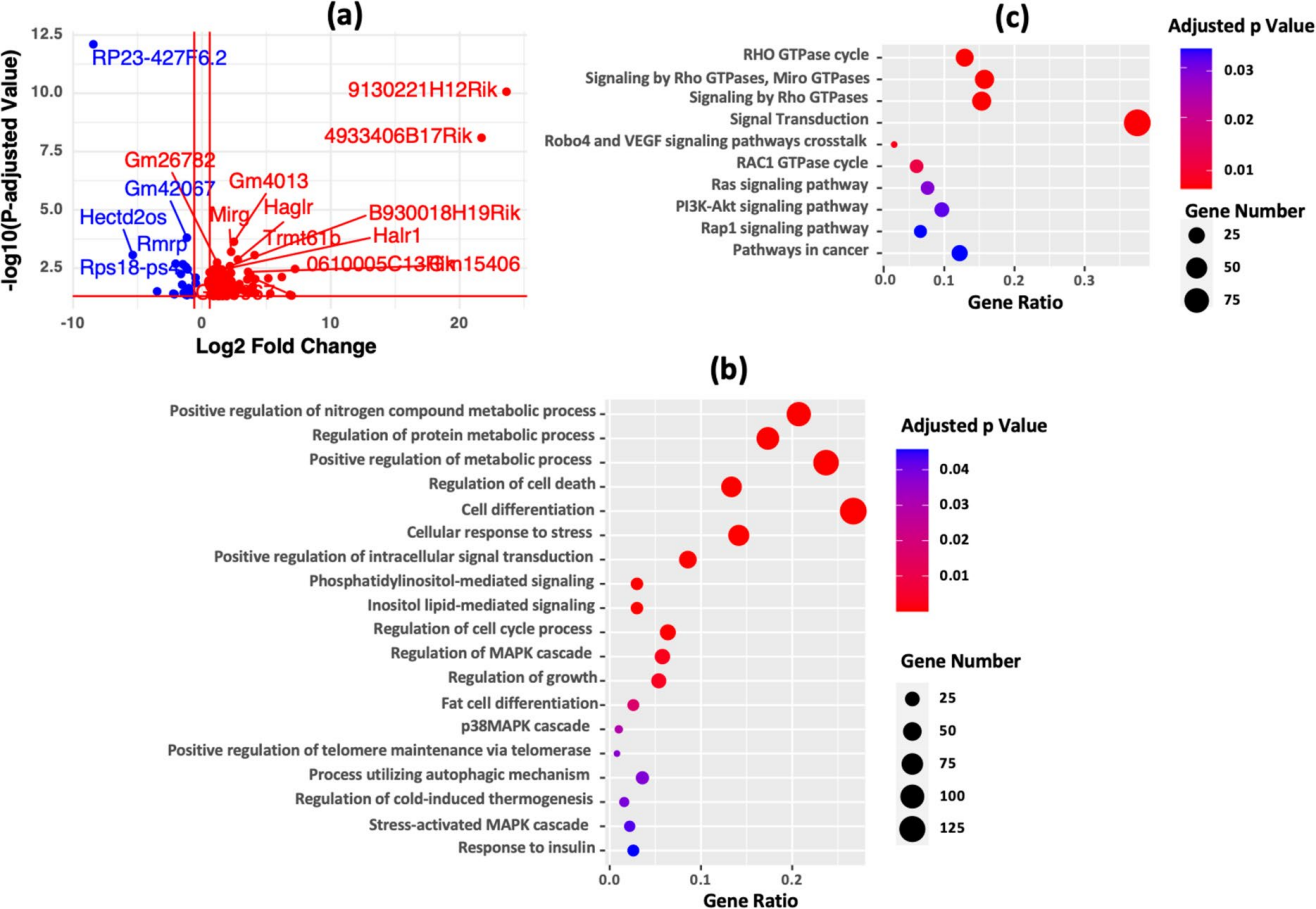
Differentially expressed lncRNAs and their potential target pathways. **a** Long non-coding RNA (lncRNA) differential expression volcano plot. The plot presents the differential expression levels of lncRNA genes. Upregulated lncRNAs are indicated by red dots, while downregulated lncRNAs are marked with blue dots. **b–c** Enriched pathway analysis of targeted protein-coding genes. Pathway enrichment was performed on genes potentially targeted by the differentially expressed lncRNAs. The analysis utilized the gprofiler2 tool with “fdr” correction to assess significance, applying a threshold of *p* < 0.05. Pathways are ordered on the *y*-axis according to their adjusted p-value, and the *x*-axis depicts the enrichment ratio—the ratio of observed to expected genes within each pathway. The size of each dot reflects the number of genes contributing to each pathway (intersection size), and the color intensity represents the significance level of the adjusted *p*-values. This analysis encompasses Gene Ontology biological processes (**b**) and pathways from databases such as KEGG and REACTOME (**c**)

We enhanced our study through the analysis of long non-coding RNAs (lncRNAs) and identified 132 lncRNAs with significant differential expression (Fig. 4a and Table S10). By using the RNAInter database for functional predictions [37], we identified the protein-coding genes targeted by these lncRNAs (Table S11). Using gprofiler2 [36] for enrichment analysis, we found that these lncRNAs influence Gene Ontology pathways related to metabolism and many cellular functions (Fig. 4b and Table S12). Moreover, the analysis highlighted the involvement of these lncRNAs in KEGG and REAC pathways, including critical pathways such as Ras and PI3K-Akt, which are important for cell survival, growth, and metabolic processes (Fig. 4c). Pathways involving Rap1 and Rho GTPase, important for tissue repair, may also play a role in lowering cancer risks and contributing to the observed longevity in df/df mice.

### Applying WGCNA uncovers co-expression networks of lncRNAs in df/df adipose tissue

**Fig. 5:**
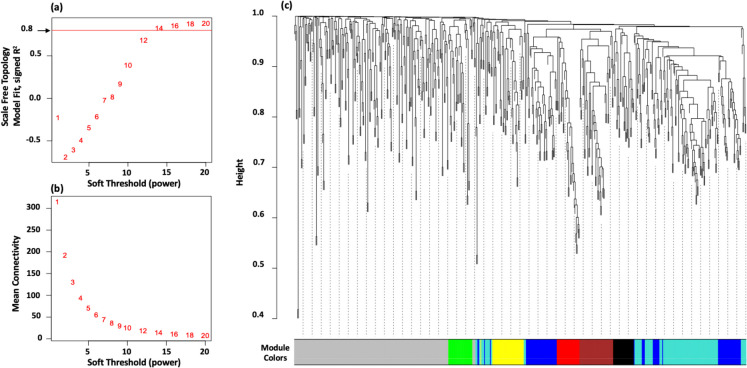
Selection of soft-thresholding power for WGCNA**. a** Scale independence analysis. The graph evaluates scale independence, indicating how well the network conforms to a scale-free topology—essential for biological network construction. The soft-thresholding power is plotted on the *x*-axis against the scale-free fit index on the *y*-axis, where higher signed *R*^2^ values suggest a strong adherence to scale-free properties. The goal is to determine a power value that assures the network is not random but inherently scale-free. **b** Mean connectivity assessment. The graph depicts the average number of connections per node, providing insight into the network’s interconnectivity. The soft-thresholding power is plotted on the *x*-axis against the mean connectivity on the *y*-axis. Optimal soft-thresholding power is chosen to minimize mean connectivity while retaining a high scale independence, indicative of a scale-free network. A power of 16 is identified as optimal in this instance, corresponding to a scale-free fit index near 0.80 and a low mean connectivity. **c** Gene module hierarchical clustering. The dendrogram displays the results of hierarchical clustering, identifying modules of co-expressed genes via WGCNA. The *y*-axis measures the height at which genes converge, indicating similarity in expression. Each color band represents a module, grouping genes by expression pattern, with grey denoting genes unclassified into modules. The dendrogram facilitates the identification of gene modules potentially related to the df/df phenotype

**Fig. 6:**
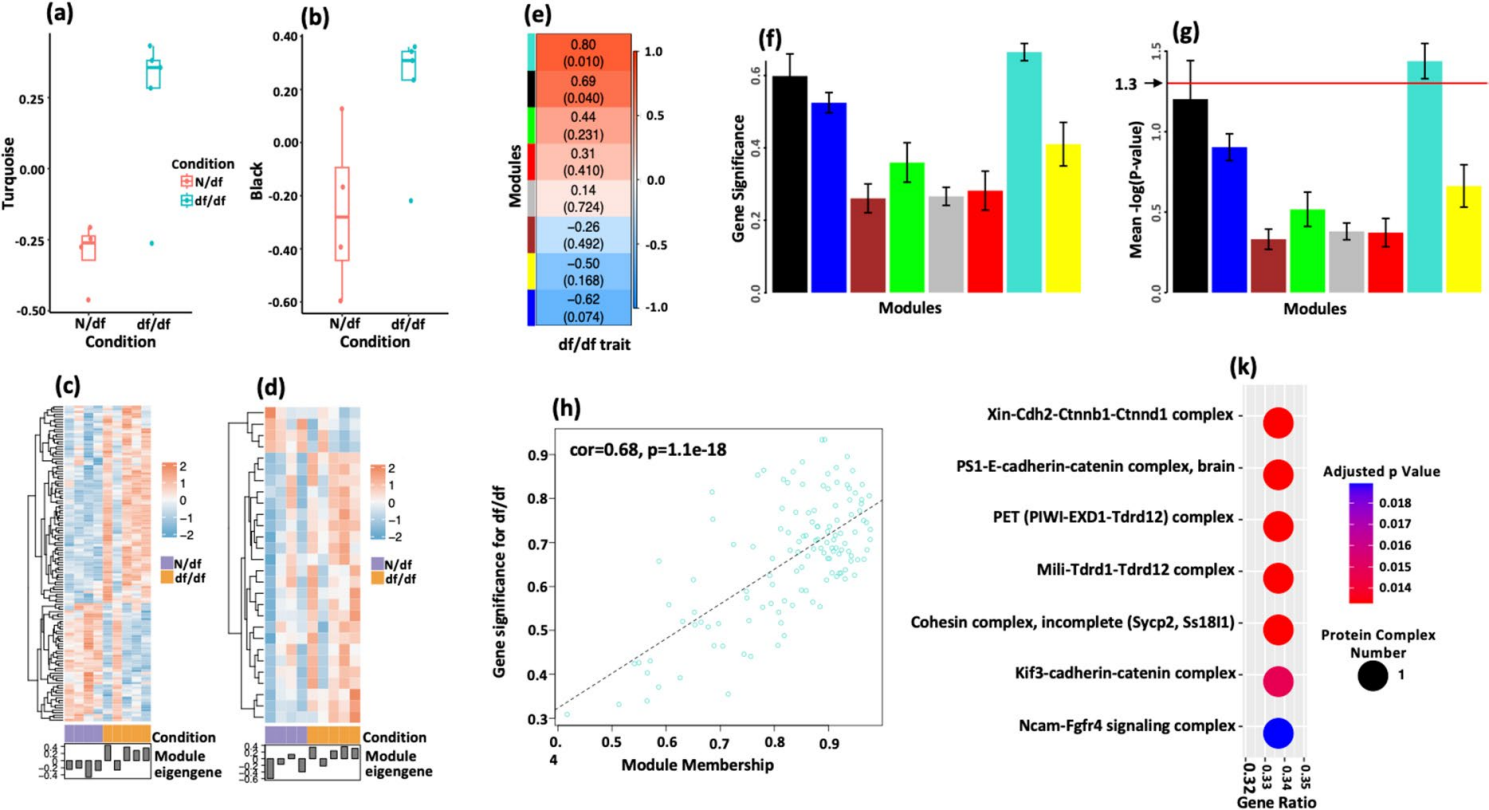
Module eigengene analysis and gene module correlation. **a–b** Eigengene value distribution for normal (N/df) vs. df/df conditions. Boxplots compare the distributions of eigengenes for the turquoise (**a**) and black (**b**) modules. Eigengenes are vectors that represent a module’s gene expression patterns, and they are instrumental for associating module activity with the df/df trait. These boxplots show the median, quartiles, and range of eigengene expressions, revealing differences between N/df and df/df samples. **c–d** Gene expression heatmaps for turquoise and black modules. These heatmaps show gene expression patterns within the turquoise (**c**) and black (**d**) modules when comparing N/df to df/df mice. Rows symbolize individual genes; columns correspond to sample types. The color intensity reflects expression levels: red indicates higher expression, blue indicates lower, and white designates intermediate expression. Hierarchical clustering on the side categorizes genes into clusters based on expression similarities. Below the heatmaps, the eigengene expression levels for each module are charted as a summary measure, comparing N/df and df/df conditions, where the *y*-axis signifies expression level and the *x*-axis differentiates the sample groups and the corresponding module eigengenes. N/df refers to mice that are heterozygous for the df allele, exhibit a normal phenotype, and are used as littermate controls for the dwarf mice (df/df). **e** Module-trait relationship heatmap. The graph illustrates correlations between gene modules and the df/df trait. Each row represents a module, colored according to its correlation with the df/df trait—red for positive and green for negative. Each cell contains the correlation coefficient and *p*-value, expressing the association’s strength and statistical significance, respectively. **f** Gene significance across modules. The bar graph quantifies the average gene significance within each module in relation to the df/df trait. Colors on the *x*-axis identify modules; the *y*-axis denotes gene significance, with taller bars indicating modules containing genes more closely associated with the trait. **g** Mean -log(*P*-value) for module gene significance. The graph plots modules along the *x*-axis, differentiated by color, against the mean -log(*P*-value) on the *y*-axis, reflecting the statistical weight of gene significance. The red horizontal line marks the significance threshold -log(*P*-value) = 1.3, corresponding to a *p*-value of 0.05. Values above this line indicate significant gene-module relationship. **h** Correlation of module membership with gene significance. A scatter plot depicts each gene’s module membership (*x*-axis) against its gene significance (*y*-axis) within the turquoise module. The correlation coefficient (cor) and *p*-value are given, indicating the strength and significance of the association. The positive correlation (cor = 0.68) indicates that genes with higher membership in the turquoise module are more significantly associated with the df/df trait, a conclusion strongly supported by the extremely low *p*-value (*p* = 1.1e − 18). A dashed trendline visualizes the correlation’s direction and strength. **k** Pathway enrichment of protein-coding genes co-expressed with LncRNAs. Pathway enrichment for protein-coding genes co-expressed with selected lncRNAs (Carmn, KCNQ1, 4930578M01Rik, Gm46404) is presented, using gprofiler2 and the CORUM database. The *y*-axis ranks pathways by adjusted *p*-value, while the *x*-axis shows the enrichment ratio. Dot size indicates the number of enriched protein complexes per pathway, with the color gradient conveying the adjusted *p*-value’s significance

To understand the role of lncRNAs in df/df adipose tissue, we used weighted gene co-expression network analysis (WGCNA) [35] to construct co-expression networks and associate gene modules with phenotypic traits, considering the well-known co-regulation of lncRNAs and mRNAs [38]. By optimizing the soft-thresholding power to achieve a scale-free topology, a value of 16 was chosen based on scale independence and mean connectivity plots, ensuring network robustness with a signed *R*^2^ above 0.80 (Fig. 5a, b). Hierarchical clustering dendrogram network analysis revealed eight distinct co-expressed gene modules (Fig. 5c). Using the Limma linear model, the study assessed gene activity differences in modules between control (N/df) and df/df groups to identify those significantly correlated with the df/df phenotype. The turquoise (Fig. 6a) and black (Fig. 6b) modules showed higher eigengene values in df/df, indicating a strong genetic influence on their expression. Heatmap analysis revealed a distinct expression profile for the turquoise module (Fig. 6c), highlighting potential regulatory gene clusters, while the black module (Fig. 6d) showed a less pronounced pattern. These results offer insights into the expression dynamics and potential impact of these modules on the df/df phenotype.

WGCNA analysis further revealed that the turquoise module has a strong positive correlation (*r* = 0.80, *p* = 0.010) with the df/df trait, stronger than the black module (*r* = 0.69, *p* = 0.040) as shown in Fig. 6e. This suggests the turquoise module more significantly influences the df/df trait. While both modules had high average gene significance (Fig. 6f), only the turquoise module’s mean -log(*P*-value) exceeded the threshold of 1.3 (Fig. 6g), indicating statistical relevance. Moreover, we found a robust positive correlation (correlation coefficient = 0.68) with highly significant *p*-value (*p* = 1.1e − 18) between module membership and gene significance in the turquoise module (Fig. 6h). This finding underscores a strong link with the df/df trait, indicating that the genes within the turquoise module are likely crucial in contributing to the characteristics of df/df.

### Identifying key lncRNAs interacting with protein complexes in df/df adipose tissue

We used rigorous selection criteria to identify significant genes within the turquoise module, requiring a |log2FC|> 0.49, an FDR < 0.05, and *p*-values < 0.05. Additionally, genes had to exhibit a gene significance and module membership with *p*-values < 0.05. This approach led to the identification of four lncRNAs—including Carmn and Kcnq1ot1—which interact with 29 protein-coding genes (Table S13). According to the “guilt by association” principle [39], it is suggested that these lncRNAs, along with their protein-coding gene partners, are involved in the same biological pathways.

Pathway enrichment analysis of co-expressed protein-coding genes, using gprofiler2, revealed their involvement in seven protein complexes (Table S14 and Fig. 6k). The complexes, comprising proteins like Cdh2, Piwil2, and Sycp2, were retrieved from the CORUM database [40]. Comprehending the structure and changes of these protein complexes is key to elucidating their contribution to the health and longevity of df/df mice and could provide new insights into molecular aging mechanisms.

## Discussion and conclusion

### Gene and transcript-level analysis identifies adipose tissue pathways associated with enhanced health and longevity in df/df mice

In our study, comprehensive analysis uncovered subtle yet meaningful changes in gene expression. These include genes displaying transcript changes while maintaining unchanged overall expression levels. Such findings reveal critical biological alterations and assist in the discovery of novel mRNA variants and splicing modalities, thereby advancing understanding of gene regulation [41]. Functional analysis indicated that the 876 genes with unchanged overall expression but differentially expressed transcripts are involved in critical regulatory pathways such as cell cycle, stress response, apoptosis, growth, and MAPK signaling (Figure S1a. These pathways are essential for cell proliferation, differentiation, and stress response. Specifically, the MAPK pathway, which includes ERK1/2, JNK, and p38 MAPK, is integral to cellular growth and differentiation; ERK1/2 is vital for cell cycle and proliferation [42], while p38 MAPK and JNK are crucial for oxidative stress response and can induce apoptosis depending on stress levels [43].

Gene expression alterations at the gene and transcript levels are linked to cell adhesion and signaling pathways, impacting cellular functions and immune responses (Figures S1b, c, d) [44]. Integrins play a critical role in cell interactions, with effects on cellular activation and gene expression, and are crucial for immune defense and inflammatory disease onset. Imbalances in integrin-mediated cytokine production can lead to chronic inflammation [45, 46]. These changes may enhance tissue integrity and immune responses in mice, potentially improving health and lifespan.

Pathways involving genes from all expression groups are crucial for metabolic processes, including protein and nutrient metabolism (Fig. 1f), with key metabolites modulating gene regulation, proliferation, and immunity [47]. Nitrogen and phosphorus metabolism are essential for physiological functions and are implicated in diseases related to metabolism like obesity and diabetes [48, 49]. Balanced metabolic processes are vital for preventing disease and may explain the enhanced health and lifespan of df/df mice through improved metabolic regulation.

Classifying genes by expression at the gene, transcript, or both levels indicates distinct pathway enrichments and a link between expression patterns and cellular functions. Comprehensive analysis of both gene and transcript levels is critical for a complete understanding of gene regulation. The identified pathways, without overall gene expression changes, suggest further research is needed to explore their effects on health and longevity in the df/df phenotype.

### Alternative splicing as a potential driver of functional consequences arising from isoform switching within genes with unchanged expression

Our study of df/df mice identified transcriptional complexity, with 124 isoforms from 100 genes showing significant isoform switches but no change in overall gene expression. Of these, 65 genes showed 70 isoform pairs with functional changes in protein domains and other molecular features. Switched isoforms mainly showed domain loss, aligning with IDR characteristics, and had increased intron retention with shorter overall ORF lengths.

Protein domains are structural segments of proteins with autonomous functions and engage in critical molecular interactions. Changes in these domains can significantly influence protein performance and biological outcomes, potentially affecting health and disease [50–52]. In df/df mice, such domain modifications may promote protein stability and enhance molecular interactions, possibly leading to increased resilience and longevity.

IDRs are flexible protein segments crucial for adaptive cellular signaling [53]. They are linked to disease when their regulation fails, contributing to neurodegenerative conditions [54, 55], heart disease [56], and cancer [57]. IDRs adjust their function under stress, aiding in cellular adaptation [58, 59]. In aging mice, this adaptability of IDRs may help maintain cell function and promote disease resistance, aligning with the health and longevity seen in df/df mice.

IR can cause translation changes and mRNA degradation, influencing gene expression and disease development, such as cancer [60, 61]. Increased IR in aging organisms points to its role in mRNA processing and protein homeostasis, suggesting it may contribute to diseases like Alzheimer’s [62, 63]. IR’s prevalence in disease indicates a possible role in lifespan and health regulation in df/df mice.

NMD regulates transcripts with premature termination codons and serves as a broader regulatory system [64], influenced by stressors like ROS and nutrient shortage [65–67]. NMD inhibition stabilizes specific transcripts, enhancing stress responses, such as with ATF4 mRNA, increasing resistance to endoplasmic reticulum stress [68]. Oncogenes, like c-myc, interfere with NMD through pathways involving PERK kinase [66, 69], which could be key in cancer gene regulation. The effects of NMD and isoform switching in df/df mice, especially on stress-response mRNAs, warrant further study.

Intriguingly, our study finds that genes with unchanged expression levels undergoing isoform switching are engaged in alternative splicing, including ATSS, ATTS, and AS, leading to structural intron variations (Fig. 3b, Table S8). Such splicing events relate to functional changes like protein domain alterations and ORF length variations. These findings suggest that alternative splicing plays a role in isoform switching with potential links to the improved healthspan and longevity in df/df mice, though further research is needed to understand the mechanisms and implications fully.

### Isoform switching could be key to *cancer* resistance and metabolic health, enhancing longevity in df/df mice

Isoform switching in df/df mice suggests significant gene expression alterations with cancer implications. For instance, Arhgef4’s isoform change could affect APC tumor suppressor interaction [70], influencing cancer cell behavior and potentially df/df mice’s lifespan. PHF3’s dominant isoform absence and a minor isoform’s retention of important domains may relate to glioblastoma survival [71]. Zfp607a’s NMD-sensitive preferred isoform might lower a protein involved in hepatocellular carcinoma, possibly reducing tumor incidence in df/df mice [72].

Our study found metabolic genes in df/df mice like Slc25a25, Dot1l, and Snap47 with significant isoform changes. Slc25a25’s altered isoform may impact mitochondrial function and obesity resistance [73–75]. Dot1l’s isoform switch could disrupt lipid biosynthesis, affecting cardiovascular health [76], while Snap47’s switch might be important for lipid metabolism and longevity [77].

Finally, the pseudogene Mid1-ps1 exhibits an isoform switch from being NMD-prone to having an extended ORF with new functional domains, indicating potential unexpected protein-coding functions in df/df mice.

These findings highlight the importance of isoform switching analysis in uncovering the molecular mechanisms underlying aging and longevity in df/df mice. Isoform switching can significantly impact protein function by altering key regulatory domains involved in essential processes such as signal transduction, cell cycle control, and DNA repair. These processes are closely linked to tumor suppression and metabolic regulation, both of which are crucial for maintaining cellular homeostasis during aging [78]. For example, isoform switches that affect protein domains may lead to loss of function, altered localization, or modified interactions. This can impact processes such as adipogenesis, insulin signaling, or inflammation—factors relevant to aging-related metabolic disorders [79].

Intron retention, another consequence of isoform switching observed in this study, can produce truncated proteins or affect mRNA stability, both of which can significantly alter cellular function [62]. Specifically, intron retention in metabolic regulators may influence lipid metabolism or energy balance, pathways critical to aging and longevity [80, 81]. For instance, intron retention could lead to the production of non-functional proteins that disrupt normal metabolic processes, thereby affecting the organism’s overall energy homeostasis.

Further research is needed to explore how expression changes in metabolic and cancer-related genes may enhance cancer resistance and metabolic efficiency, potentially contributing to the health and longevity of df/df mice.

### LncRNAs are potential key regulators of metabolic and genetic stability pathways in df/df mice

Incorporating lncRNAs into our differential expression analysis has identified 132 lncRNAs with significant expression changes. These may regulate essential biological pathways, potentially enhancing metabolism, cell signaling, and stress response and reducing DNA replication errors, thereby possibly lowering cancer risks and contributing to the longevity of df/df mice [82–87].

lncRNAs target pathways crucial for cell survival, metabolism, and tissue repair, such as Ras and PI3K-Akt, potentially reducing DNA errors and cancer risk. They regulate gene expression, affecting chromatin and diseases like cancer and neurodegeneration [88–92], and influence aging processes via genes like p53 and p16INK4a [93, 94]. The role of lncRNAs in conferring resistance to aging and disease in df/df mice requires further investigation. The lncRNAs identified in this study are hypothesized to modulate metabolic processes and influence aging by regulating transcription and serving as scaffolds for chromatin-modifying complexes, as supported by previous literature [95–98]. This regulatory role is in line with findings that lncRNAs can affect chromatin dynamics and gene expression, contributing to cellular homeostasis.

Additionally, lncRNAs have been shown to influence lipid and carbohydrate metabolism as well as insulin sensitivity—critical for maintaining metabolic balance during aging [99–102]. The differential expression of these lncRNAs is likely to impact genes associated with energy metabolism, insulin signaling, and inflammatory responses [103], which are key pathways in the aging process and metabolic disorders. Prior research has demonstrated that lncRNAs can modulate metabolic pathways, thereby influencing susceptibility to age-related diseases such as diabetes and obesity [103–105]. Moreover, lncRNAs may play a crucial role in regulating immune responses [106–108], which are integral to the progression of aging and related diseases, including insulin resistance and cancer [109–111]. These findings align with previous studies that emphasize the involvement of lncRNAs in immune regulation and inflammation during aging.

To build upon these hypotheses, future research should focus on functional validation of these lncRNAs through knockdown or overexpression studies, which will allow us to assess their specific impact on metabolic gene expression and cellular processes in adipose tissue. Additionally, integrating data from other regulatory mechanisms, such as LncRNA-mRNA co-expression and protein-RNA interactions, could provide a more comprehensive understanding of how lncRNAs contribute to aging and metabolic regulation. By exploring these pathways in greater depth, we aim to uncover the molecular mechanisms through which lncRNAs influence resistance to aging and metabolic diseases..

### Adipose tissue lncRNAs are associated with protein complexes that may improve health and longevity in df/df mice

Our study applied WGCNA, identifying the turquoise module with a strong df/df trait correlation (*r* = 0.80), suggesting a significant association. Four lncRNAs in this module—Carmn, Kcnq1ot1, 4930578M01Rik, and Gm46404—interacted with 29 protein-coding genes linked to seven protein complexes. Key genes like Cdh2, Piwil2, and Sycp2 were implicated, with references from the CORUM database [40]. Understanding these protein complexes is critical to uncover their contribution to the health and longevity of df/df mice.

The Mili-Tdrd1-Tdrd12 and PET (PIWI-EXD1-Tdrd12) complexes are key in producing piRNAs that suppress transposons, ensuring fertility and genomic stability [97, 98, 112]. piRNAs in testes and ovaries protect genomes, with ovarian piRNAs varying with age and the df/df genotype, indicating a role in longevity [14]. They also partake in ovarian cancer regulatory networks [113] and extend functions to somatic and stem cells, influencing gene expression, chromatin formation, and neuronal development, with dysfunctions linked to neurodevelopmental disorders [114, 115]. In Drosophila, piRNA pathways are vital for lipid metabolism and overall health [116]. Our research shows similar piRNA pathway activity in df/df mice, suggesting a possible impact on metabolic health that merits further study.

The NCAM-FGFR4 signaling complex modulates FGFR-related NCAM signaling, impacting cellular processes including calcium regulation and neural signaling, with consequences for brain health and disease [117–120]. The cohesin complex, comprising proteins like Sycp2 and Ss18I1, is critical for DNA repair and maintenance of genomic stability, as well as gene expression and chromatin architecture, playing a vital role in development and cancer pathology [121–125].

Cadherin complexes, crucial for cell adhesion, support organ, and tissue structure, influence proliferation and communication pathways crucial for tissue health [126]. Disruptions in these complexes, particularly the E-cadherin-catenin assembly, are linked to diseases like inflammatory bowel disease [127], and they are also key players in cancer progression, underscoring their importance in health and disease.

Modifications in NCAM-FGFR4, cohesin, and cadherin complexes in df/df mice adipose tissue may play roles in their longevity. The NCAM-FGFR4 complex may improve brain health and cognitive function. Cohesin role in DNA repair and gene expression could prevent diseases and preserve stem cell health. Cadherins involvement in tissue integrity could protect against degenerative conditions. Collectively, these complexes might contribute to df/df mice’s extended healthspan.

## Conclusion

The differential expression of coding genes, lncRNAs, and isoform switches identified in df/df mice may play critical roles in metabolic regulation and aging. This study provides insights into how genetic factors influence adipose tissue function, which is strongly linked to age-related metabolic diseases such as obesity, insulin resistance, and type 2 diabetes. Importantly, the identified isoform switches have translational potential, as they may serve as biomarkers or therapeutic targets. Future research could explore these targets to develop interventions aimed at modulating adipose tissue function, improving metabolic health, and potentially extending healthspan. Additionally, this study lays the groundwork for further investigation into the functional consequences of isoform switching. Future directions could involve protein-level validation to assess the impact of isoform switches on protein function and interaction networks, functional studies to determine the physiological effects of modulating these isoforms, and cross-species comparisons with human data to identify conserved pathways relevant to human health and aging.

## Supplementary Information

Below is the link to the electronic supplementary material.

Supplementary file1 (DOCX 4758 KB)

Supplementary file2 (XLSX 807 KB)

## Data Availability

The data that support the findings of this study are available in the supplementary material of this article.
