# Supplementary material for: Gene and transcript expression patterns, coupled with isoform switching and long non-coding RNA dynamics in adipose tissue, underlie the longevity of Ames dwarf mice
Source: GeroScience. 2024 Oct 15;47(2):1923–43. doi: 10.1007/s11357-024-01383-x (PMC11978586; doi:10.1007/s11357-024-01383-x)
Supplement: Supplementary file 1 — Supplementary file1 (DOCX 4758 KB) [file 11357_2024_1383_MOESM1_ESM.docx]

**This document contains Supplementary Figures S1-S7.**

**
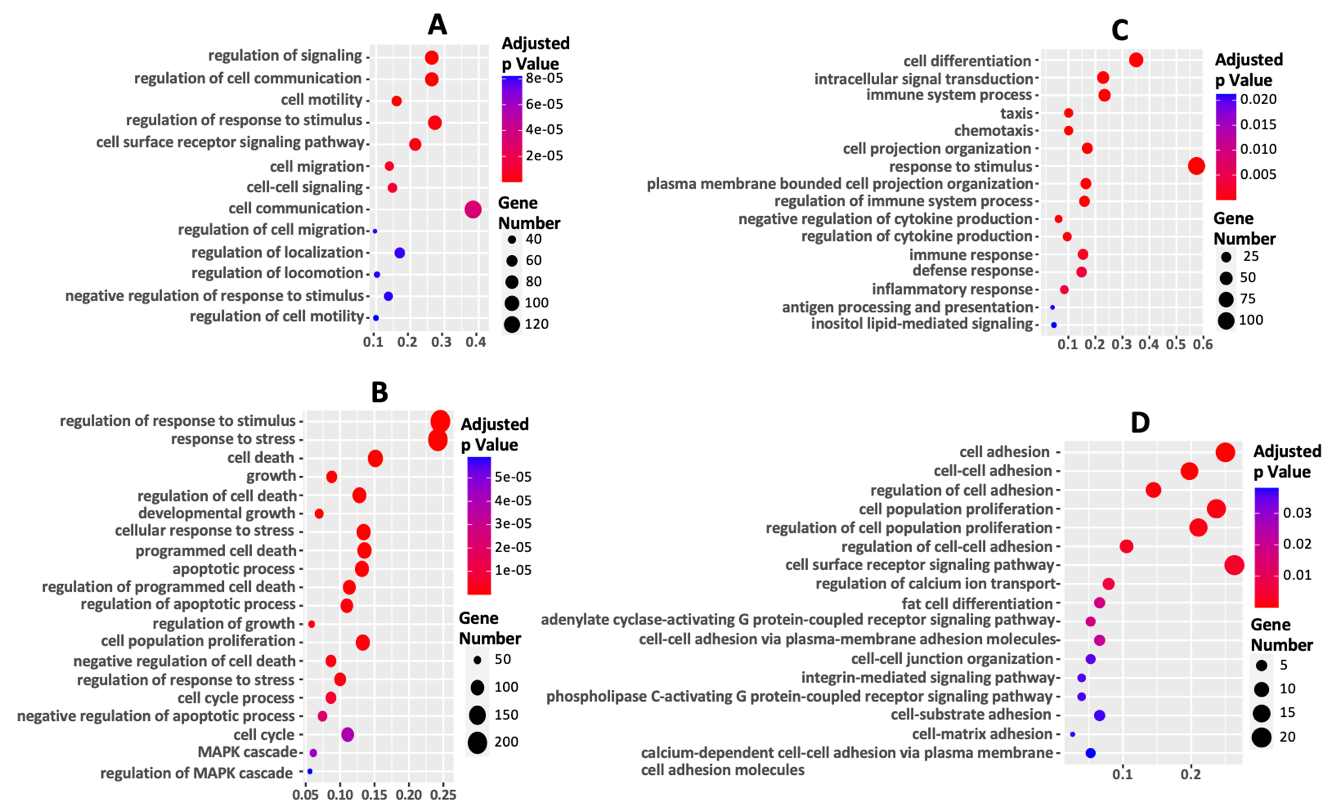
**

**Figure S1: GO Biological Process Enrichment Across Gene Expression Categories in df/df Mice Adipose Tissue.**

This graph presents the enrichment of Gene Ontology (GO) Biological Processes as identified in the adipose tissue gene expression profiles of df/df mice, relating to Figure 1. Enrichment was assessed using the gprofiler2 tool with an "fdr" correction method and a p-value threshold set below 0.05 for significance. Displayed are the selected biological terms that are non-redundantly enriched across four expression categories: **(A)** genes with expression changes exclusive to the gene level; **(B)** genes with expression changes unique to the transcript level; **(C)** genes with expression changes at both gene and transcript levels; **(D)** genes with expression changes at the gene level and concurrent changes in a subset of their transcripts. Pathways are listed on the y-axis, organized by the significance of their adjusted p-value. The x-axis indicates the enrichment ratio, defined as the observed over expected gene count within a pathway. The size of each dot reflects the quantity of genes contributing to each term, known as the intersection size, while the color gradient denotes the statistical significance of the term's adjusted p-value. N/df refers to mice that are heterozygous for the df allele, exhibit a normal phenotype, and are used as littermate controls for the dwarf mice (df/df).

**Figure S2. Isoform switching in the PHF3 gene.**

**(A)** Structural representation of PHF3 gene isoforms. Coding exons are represented by blocks connected by horizontal lines representing introns. The 5’ and 3’ UTRs are represented by thinner blocks at the beginning and end of the transcript. Colored boxes indicate predicted protein domains. Ensembl gene transcript IDs are shown for each isoform. MSTRG are names of novel isoforms.

**(B)** Graphical analysis of PHF3 gene expression, showing the unchanged overall gene expression (left) with isoform-specific expression alterations (middle), and the differential usage of a switched-up and switched-down isoform pair (right). ‘ns’, not significant; ‘*’, adjusted p-value < 0.05. N/df refers to mice that are heterozygous for the df allele, exhibit a normal phenotype, and are used as littermate controls for the dwarf mice (df/df).

**Figure S3. Isoform switching in the Zfp607a gene.**

**(A)** Structural representation of Zfp607a gene isoforms. Coding exons are represented by blocks connected by horizontal lines representing introns. The 5’ and 3’ UTRs are represented by thinner blocks at the beginning and end of the transcript. Colored boxes indicate predicted protein domains. Ensembl gene transcript IDs are shown for each isoform. MSTRG are names of novel isoforms.

**(B)** Graphical analysis of Zfp607a gene expression, showing the unchanged overall gene expression (left) with isoform-specific expression alterations (middle), and the differential usage of a switched-up and switched-down isoform pair (right). ‘ns’, not significant; ‘***’, adjusted p-value < 0.001; ‘*’, adjusted p-value < 0.05. N/df refers to mice that are heterozygous for the df allele, exhibit a normal phenotype, and are used as littermate controls for the dwarf mice (df/df).

**Figure S4. Isoform switching in the Slc25a25 gene.**

**(A)** Structural representation of Slc25a25 gene isoforms. Coding exons are represented by blocks connected by horizontal lines representing introns. The 5’ and 3’ UTRs are represented by thinner blocks at the beginning and end of the transcript. Colored boxes indicate predicted protein domains. Ensembl gene transcript IDs are shown for each isoform.

**(B)** Graphical analysis of Slc25a25 gene expression, showing the unchanged overall gene expression (left) with isoform-specific expression alterations (middle), and the differential usage of a switched-up and switched-down isoform pair (right). ‘ns’, not significant; ‘***’, adjusted p-value < 0.001; ‘*’, adjusted p-value < 0.05. N/df refers to mice that are heterozygous for the df allele, exhibit a normal phenotype, and are used as littermate controls for the dwarf mice (df/df).

**Figure S5. Isoform switching in the Dot1l gene.**

**(A)** Structural representation of Dot1l gene isoforms. Coding exons are represented by blocks connected by horizontal lines representing introns. The 5’ and 3’ UTRs are represented by thinner blocks at the beginning and end of the transcript. Colored boxes indicate predicted protein domains. Ensembl gene transcript IDs are shown for each isoform. MSTRG are names of novel isoforms.

**(B)** Graphical analysis of Dot1l gene expression, showing the unchanged overall gene expression (left) with isoform-specific expression alterations (middle), and the differential usage of a switched-up and switched-down isoform pair (right). ‘ns’, not significant; ‘***’, adjusted p-value < 0.001. N/df refers to mice that are heterozygous for the df allele, exhibit a normal phenotype, and are used as littermate controls for the dwarf mice (df/df).

**Figure S6. Isoform switching in the Snap47 gene.**

**(A)** Structural representation of Snap47 gene isoforms. Coding exons are represented by blocks connected by horizontal lines representing introns. The 5’ and 3’ UTRs are represented by thinner blocks at the beginning and end of the transcript. Colored boxes indicate predicted protein domains. Ensembl gene transcript IDs are shown for each isoform. MSTRG are names of novel isoforms.

**(B)** Graphical analysis of Snap47 gene expression, showing the unchanged overall gene expression (left) with isoform-specific expression alterations (middle), and the differential usage of a switched-up and switched-down isoform pair (right). ‘ns’, not significant; ‘*’, adjusted p-value < 0.05. N/df refers to mice that are heterozygous for the df allele, exhibit a normal phenotype, and are used as littermate controls for the dwarf mice (df/df).

**Figure S7. Isoform switching in the Mid1-ps1 gene.**

**(A)** Structural representation of Mid1-ps1 gene isoforms. Coding exons are represented by blocks connected by horizontal lines representing introns. The 5’ and 3’ UTRs are represented by thinner blocks at the beginning and end of the transcript. Colored boxes indicate predicted protein domains. Ensembl gene transcript IDs are shown for each isoform. MSTRG are names of novel isoforms.

**(B)** Graphical analysis of Mid1-ps1 gene expression, showing the unchanged overall gene expression (left) with isoform-specific expression alterations (middle), and the differential usage of a switched-up and switched-down isoform pair (right). ‘ns’, not significant; ‘*’, adjusted p-value < 0.05. N/df refers to mice that are heterozygous for the df allele, exhibit a normal phenotype, and are used as littermate controls for the dwarf mice (df/df).
